# Increased expression of METTL3 in pancreatic cancer tissues associates with poor survival of the patients

**DOI:** 10.1186/s12957-022-02743-7

**Published:** 2022-09-05

**Authors:** Yuan Li, Hao Huang, Yulan Zhu, Bin Xu, Junjun Chen, Yingting Liu, Xiao Zheng, Lujun Chen

**Affiliations:** 1 Department of Tumor Biological Treatment, The Third Affiliated Hospital of Soochow University, Changzhou, 213003 People’s Republic of China; 2 Jiangsu Engineering Research Center for Tumor Immunotherapy, The Third Affiliated Hospital of Soochow University, Changzhou, 213003 People’s Republic of China; 3 Institute of Cell Therapy, The Third Affiliated Hospital of Soochow University, Changzhou, 213003 People’s Republic of China

**Keywords:** METTL3, Pancreatic cancer, Immunohistochemistry, Prognosis

## Abstract

**Background:**

Methyltransferase-like 3 (METTL3) expression could be found in various normal and cancerous tissues. As of now, the clinical significance of METTL3 expression in human pancreatic cancer (PC) tissues still remains to be understood. Our present study aims to investigate the prognostic value and clinical implications of METTL3 expression in PC tissues.

**Methods:**

The TCGA, GTEx, and GEO public databases were used to study the mRNA expression level of the m^6^A family members and its relationship among PC tissues and normal pancreatic tissue. The immunohistochemistry was used to analyze the difference of METTL3 expression between cancer tissues and adjacent normal tissues. The prognostic value was evaluated by using the Log-rank survival analysis and Cox model analysis. PAAD samples from TCGA and GEO databases were used to perform the immune infiltration analysis and gene set enrichment analysis based on the genes that were highly correlated with METTL3.

**Results:**

Based on the analysis of TCGA, GTEx, and GEO public database, we found that the m^6^A family members showed a higher correlation in PC tissues compared to normal pancreatic tissues, and the mRNA expression level of the m^6^A family members showed a significant difference between PC tissues and adjacent normal tissues. Moreover, scRNA-seq data indicated that METTL3 showed a higher expression level in malignant epithelial cells. Our immunohistochemistry results also confirmed that the intensity of METTL3 immunostaining in PC tissues was significantly higher than that in adjacent normal tissues (*P* = 0.015). The overall survival (OS) of PC patients with high expression of METTL3 protein were significantly poorer than those with low expression of METTL3 protein (HR = 1.788, 95% CI 1.071–2.984, *P* = 0.026). Further analysis of PC data from the database showed that METTL3 expression was associated with a variety of tumor-infiltrating immune cells and was involved in m^6^A modification and metabolism in PC tissues.

**Conclusion:**

Increased METTL3 expression at the protein level could be found in PC tissues, suggesting that the METTL3 expression was involved in the progression of PC and could serve as an important marker for prognostic prediction of this malignancy.

## Introduction

Pancreatic cancer (PC) is a highly malignant tumor from digestive system with poor prognosis [1, 2]. Worldwide, PC is the twelfth most common cancer in men, and the eleventh most common cancer in women, and the seventh leading cause of cancer-related deaths [3]. The occurrence of PC is closely related to lifestyle, and smoking, alcohol, and diet are also risk factors [4]. The patients suffering from PC usually diagnosed at advanced stages, with nonspecific symptoms, or lack of sensitive and specific tumor markers, and the 5-year survival is less than 10% [2, 5–7]. Therefore, it is urgently necessary to develop novel biomarkers and therapeutic strategies for the treatment of PC [8].

m^6^A is the most prevalent internal modification in polyadenylated mRNAs and long non-coding RNAs (lncRNAs) in higher eukaryotes [9]. Generally, there were 3–5 m^6^A sites in each mRNA in the conservative sequence RRACH (R = G or A, H = A, C or U), it is enriched in distribution in the vicinity of long exon, termination codon and 3' end noncoding region (3′UTR) [10, 11]. METTL3 and methyltransferase like-14 (METTL14) are core proteins of m^6^A methyltransferase [12]. It is well-known that METTL3 plays an important role in promoting cancer proliferation in several human cancers, such as breast cancer, colorectal carcinoma, lung cancer, ovarian carcinoma, bladder cancer, and so on [13–17].

METTL3, also known as MT-A70, has two critical domains, which are used to bind S-adenosylmethionine (SAM) and to catalyze the formation of m^6^A, respectively [18, 19] METTL3, together with METTL14, could form a stable heterodimer core complex of METTL3-14 that functions in cellular m^6^A deposition on mammalian nuclear RNAs [9]. METTL3 has been shown to function as an important gene to promote tumor progression. For example, up-regulated METTL3 could promote metastasis of colorectal cancer cells through miR-1246/SPRED2/MAPK signaling pathway [20]. In gastric cancer, METTL3 was upregulated and the elevated METTL3 level was an important predictor for poor prognosis of the patients [21]. There is also evidence shows that METTL3 can accelerate cell proliferation in breast cancer via inhibiting tumor suppressor let-7 g, and METTL3 could increase HBXIP expression to form a positive feedback loop of HBXIP/let-7 g/METTL3/HBXIP [13]. METTL3 could also promote translation of certain oncogenic mRNAs, and METTL3 depletion could sensitize lung cancer cells to BRD4 inhibition and inhibit tumorigenicity [22]. In addition, we also found that METTL3 was significantly correlated with other members of the m^6^A system in PC tissues compared with normal tissues. These results suggest that METTL3 may exist as a pivotal oncogene that promotes cancer progression. As of now, the expression pattern and the clinical significance of METTL3 in PC have not been reported.

As of now, the clinical significance of METTL3 expression in human PC tissues still remains largely unexplored. Herein, in our present study, we examine the METTL3 expression in both human PC tissues and adjacent normal pancreatic tissues, and further retrospectively investigated the prognostic value and clinical implications of METTL3 expression in PC. Moreover, bioinformatics analysis was also performed to reveal the essential role of METTL3 in functional regulation of human PC tissues.

## Materials and methods

### Patients and samples

The human pancreatic cancer tissue microarray, including 99 PC tissues and 71 corresponding adjacent normal tissues, was purchased from Shanghai Outdo Biotech Co., Ltd. (Lot number: H-Pan-Ade170Sur-01). None of the patients received radiotherapy, chemotherapy or other adjuvant anti-tumor treatment before operation, and they were histo-pathologically diagnosed as PC. Due to the missing of four tissue points during antigen retrieval, and survival information missing of 4 patient samples, finally a total of 91 patients (including 55 males and 36 females), were included in the present study. The detailed clinical parameters were listed in Table 1. All the cancer tissues were confirmed as PC by H&E staining and pathological examination. Our present study was approved by the ethics committee of our hospital.

**Table 1:** Correlation between METTL3 expression and clinical parameters of the patients

Characteristics	Patients	METTL3 expression		*χ*^*2*^	*P* value
		*H *score ≤ 185	*H *score > 185		
Gender					
Male	55	23	32	0.077	0.781
Female	36	14	22		
Age(year)					
< 60	46	13	33	5.927	**0.015**
≥ 60	45	24	21		
Tumor size (diameter, cm)					
< 4	34	11	23	1.552	0.213
≥ 4	57	26	31		
TNM stage					
I	37	15	22	0.000	0.985
II + III + IV	54	22	32		
Vascular invasion					
No	54	21	33	0.173	0.678
Yes	37	16	21		
Survival time (months)					
< 11	45	17	28	0.306	0.580
≥ 11	46	20	26		
Pathological stage					
II	61	25	36	0.008	0.928
III + IV	30	12	18		

Bold signifies *P* < 0.05

### Immunohistochemistry (IHC)

IHC assay was used to examine the METTL3 expression in human PC tissues and adjacent normal tissues. Briefly, paraffin-embedded tissue chips were dried at 90 °C for 4 h, dewaxed in xylene, and then rehydrated in graded ethanol solutions. EDTA solution (1 mM, pH 9.0) was used for antigen retrieval. Cooled tissue sections were immersed in 0.3% hydrogen peroxide solution for 15 min to block endogenous peroxidase activity, rinsed with PBS for 5 min and blocked with 3% BSA solution at room temperature for 30 min. After washing by PBS, the sections were incubated with the primary antibody (rabbit anti-human METTL3 monoclonal antibody, Catalog No. ab195352, purchased from Abcam, used in 1:150) at 4 °C overnight, followed by incubation with HRP-labeled secondary antibody at 37 °C for 30 min. Diaminobenzene was used as the chromogen, and hematoxylin was used as the nuclear counterstain. Sections were then dehydrated, cleared and mounted.

### Evaluation of IHC staining

The immunostaining intensity of METTL3 was assessed according to the *H *score method as previously described [23, 24]: *H *score = (% unstained tumor cells × 0) + (% weakly stained tumor cells × 1) + (% moderately stained tumor cells × 2) + (% strongly stained tumor cells × 3). The *H *scores ranged from 0 (100% negative tumor cells) to 300 (100% strongly stained tumor cells). The scoring results were recorded and then used for further statistical analysis.

### Correlation and different expression analysis of m^6^A family

TCGA PAAD RNAseq data were downloaded from the UCSC Xena website (https://xenabrowser.net/). RNAseq data of normal pancreatic tissue was downloaded from GTEx Database. Expression profiling by an array containing PC tissues and adjacent normal tissues was downloaded from the GEO database (GEO: GSE15471). RNAseq data from TCGA and GTEx databases was used to perform a correlation analysis of the m^6^A family. Data from the GEO database was used to analyze different expression genes of the m^6^A family. The visualization of correlation and differential expression analysis were used R package “pheatmap”.

### scRNA-seq data analysis

Colon cancer and PC scRNA-seq data were downloaded from the GEO database (GEO: GSE146771; GEO: GSE155698). Downloaded data was processed by R package “Seurat” followed by the official process.

### Immune cell infiltration analysis

Scores of 24 immune infiltrating cells of TCGA PAAD were downloaded from ImmuCellAI (http://bioinfo.life.hust.edu.cn/ImmuCellAI#!/). The correlation analysis between METTL3 and immune infiltrating cells was performed by R package “ggpubr”.

### Gene set enrichment analysis

TCGA PAAD RNAseq data was used to perform the enrichment analyses based on the genes that were highly correlated with METTL3 (*r* > 0.5). Gene set enrichment analysis was performed by the R package “clusterProfiler”. Biological process, cellular component, molecular function, and KEGG were achieved with the “enrichGO” function. All plots were achieved by “dotplot” function.

### Statistical analysis

Statistical analysis was performed using Prism 7 software (GraphPad) and RStudio 6.3. Chi-square test was used to compare the disease-related factors in patients with low and high expression of METTL3 in human PC tissues. Log-rank survival analysis was used to predict the OS of patients. The Cox model was used to evaluate the prognostic values of different parameters involving METTL3 expression in PC tissues. *P* < 0.05 was considered statistically significant.

## Results

### Correlations between METTL3 and other molecules from m^6^A enzyme system

**Fig. 1:**
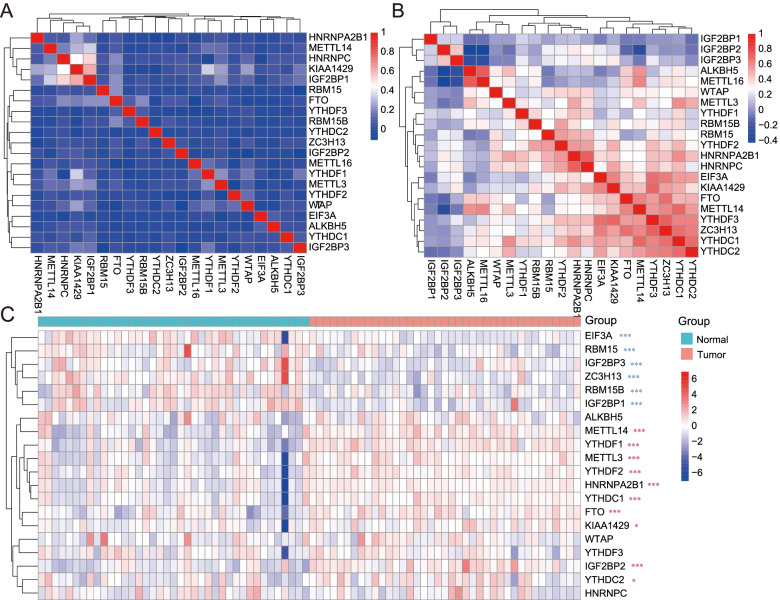
Correlation of METTL3 with other members from the m^6^A system. **A** The correlation between METTL3 and m^6^A enzyme system-related molecules in normal tissues in the GTEx database. **B** The correlation between METTL3 and m^6^A enzyme system-related molecules in PC tissues in TCGA database. **C** Different expression m^6^A enzyme system genes between normal tissues and PC tissues were analyzed in the GEO database

We analyzed RNA-seq data downloaded from GTEx and TCGA databases and found that *METTL3* expression level was significantly correlated with other molecules from m^6^A enzyme system in PC tissues compared with normal tissues (Fig. 1A, B). Higher correlation in PAAD may suggest that the m^6^A enzyme system plays an important role in tumor progression. To overcome a few samples from TCGA PAAD paired adjacent normal tissues, we analyzed 19 PC tissues and 19 adjacent normal tissues data from the GEO database. The mRNA expression level of the m^6^A enzyme system showed a significant difference between PC tissues and adjacent normal tissues (Fig. 1C). Based on the above analysis of different databases, we found that the m^6^A enzyme system showed high correlations with PC progression.

### Higher METTL3 mRNA expression level in human cancer tissues

**Fig. 2:**
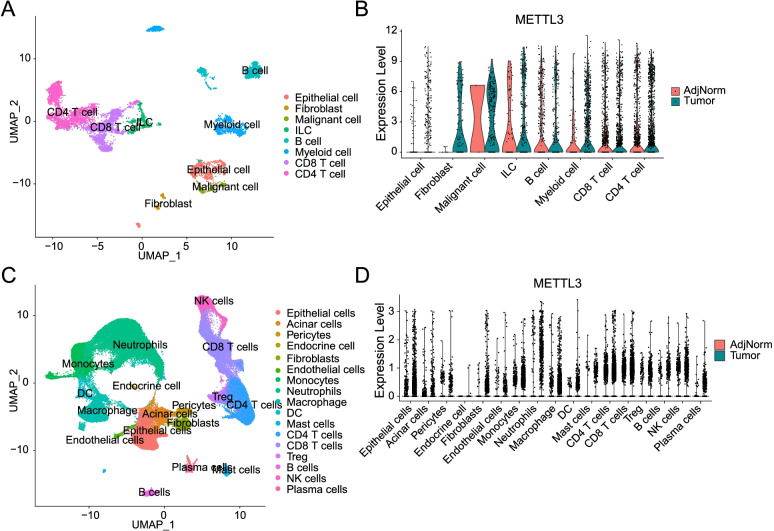
Higher METTL3 mRNA expression level in cancer tissues. METTL3 mRNA expression level between normal tissues and tumor tissues. **A**, **B** Colon cancer, GEO: GSE146771. **C**, **D**. PC, GEO: GSE155698

We then analyzed the PC and also colon cancer data downloaded from the GEO database and found that METTL3 mRNA expression level was higher in malignant epithelial cells compared with normal epithelial cells. We found that METTL3 mRNA expression level in colon cancer tissues was significantly higher than that in adjacent normal tissues (GEO: GSE146771, Fig. 2A, B). Then, we also confirmed this signature in human PC tissues (GEO: GSE155698, Fig. 2C, D). Moreover, as shown in Fig. 2, we also revealed that, in addition to the expression of METTL3 in malignant cells, it also could be found in some immune cell sub-sets. Together, METTL3, as an important member of the m^6^A enzyme system, may play an essential role in cancer progression.

### Expression and localization of METTL3 in PC tissues

**Fig. 3:**
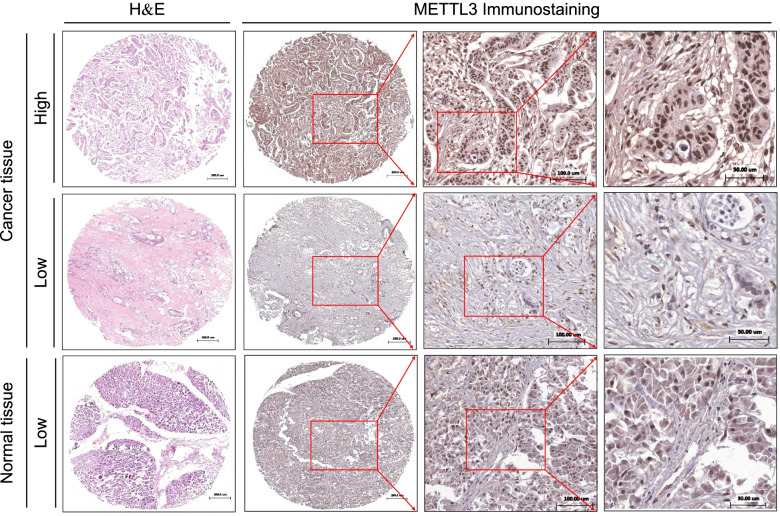
Characterization of the expression of METTL3 in human PC tissues and adjacent normal tissues by using IHC staining. **A** High expression of METTL3 in PC tissue. **B** Low expression of METTL3 in PC tissue. **C** Low expression of METTL3 in normal pancreatic tissue

**Fig. 4:**
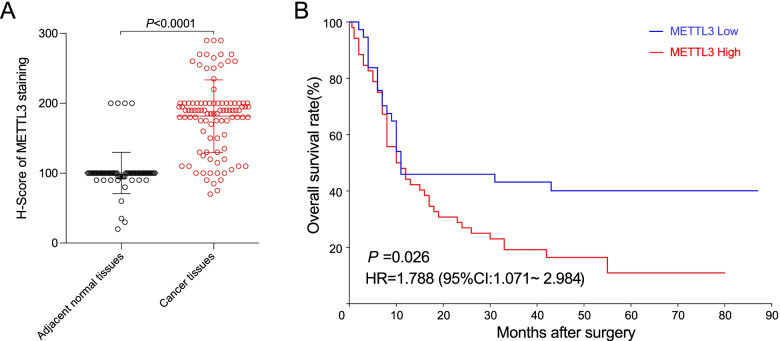
Comparison of METTL3 expression levels in PC tissues and adjacent normal tissues, and survival analysis of METTL3 expression in PC tissues. **A** The expression of METTL3 in PC tissues was significantly higher compared with normal pancreatic tissues (*P* < 0.0001). **B** Patients with low expression of METTL3 in PC tissues had a significantly better OS than those with high expression (HR = 1.788, 95% CI 1.071–2.984, *P* = 0.026)

In our present study, the METTL3 expression at the protein level in PC tissues as well as normal tissues was examined by IHC. The positive staining of METTL3 could be found mainly in the nucleus, with higher expression in cancer tissues and lower expression in adjacent normal tissues (Figs. 3 and 4A). In PC tissues, when we selected *H* score = 185 as the *cut-off* value, the patients with *H *score > 185 were assigned into the METTL3 high-expression group (54 cases), and the patients with *H *score ≤ 185 were assigned into the METTL3 low-expression group (37 cases). Table 1 shows that the increased METTL3 expression was significantly correlated with age (*χ*^2^ = 5.927, *P* = 0.015), but there were no correlations between the expression of METTL3 and gender, tumor size, TNM stage, vascular invasion, survival time, and pathological stage.

### Prognostic value of METTL3 expression in human PC tissues

Figure 4B shows that patients with high expression of METTL3 in PC tissues had a significantly worse OS than those with low expression (cut-off = 185; HR = 1.788, 95% CI 1.071–2.984, *P* = 0.026). Univariate analysis showed that the OS of patients with advanced TNM stage was significantly poorer compared with the patients with low TNM stage (HR = 1.912, 95% CI 1.149–3.181, *P* = 0.013). The OS of patients with higher expression of METTL3 was significantly poorer compared with the patients with lower expression of METTL3 (HR = 1.788, 95% CI 1.071–2.984, *P* = 0.026). The OS of patients with advanced pathological stage was significantly poorer compared with the patients with low pathological stage (HR = 1.852, 95% CI 1.127–3.043, *P* = 0.015). Multivariate Cox model analysis showed that the age (HR = 1.836, 95% CI 1.090–3.094, *P* = 0.022), TNM stage (HR = 2.407, 95% CI 1.392–4.162, *P* = 0.002), pathological stage (HR = 2.271, 95% CI 1.287–4.006, *P* = 0.005), and METTL3 expression level (HR = 2.243, 95% CI 1.309–3.843, *P* = 0.003) could be used as independent risk factors for prognosis prediction of PC patients (Table 2).

**Table 2:** Univariate and Cox model analyses of patients’ clinical parameters and METTL3 expression in PC tissues

Characteristics	Univariate analysis		Multivariate analysis	
	HR (95% CI)	*P*	HR (95% CI)	*P*
Gender (male:female)	1.126 (0.690 ~ 1.839)	0.635	1.031 (0.592 ~ 1.796)	0.914
Age (≥ 60 years: < 60 years)	1.222 (0.759 ~ 1.968)	0.410	1.836 (1.090 ~ 3.094)	**0.022**
Tumor diameter (≥ 4 cm: < 4 cm)	0.877 (0.541 ~ 1.422)	0.595	0.685 (0.407 ~ 1.152)	0.153
TNM stages (II + III + IV: I)	1.912 (1.149 ~ 3.181)	**0.013**	2.407 (1.392 ~ 4.162)	**0.002**
Distance metastasis (yes:no)	1.746 (0.426 ~ 7.162)	0.439	0.706 (0.151 ~ 3.313)	0.659
Vascular invasion (yes:no)	1.336 (0.825 ~ 2.160)	0.238	1.080 (0.649 ~ 1.798)	0.767
Pathological stage (G3 + G4:G2)	1.852 (1.127 ~ 3.043)	**0.015**	2.271 (1.287 ~ 4.006)	**0.005**
METTL3 (high:low)	1.788 (1.071 ~ 2.984)	**0.026**	2.243 (1.309 ~ 3.843)	**0.003**

Bold signifies *P* < 0.05

### Correlation analysis of METTL3 and tumor-infiltrating immune cells

**Fig. 5:**
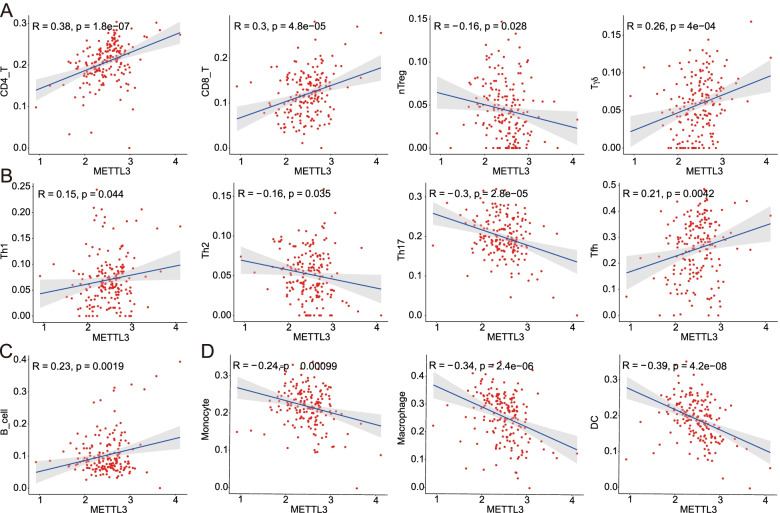
Correlation analysis of METTL3 and tumor-infiltrating immune cells. The correlation between METTL3 and tumor-infiltrating immune cells was analyzed according to the TCGA database. **A–D** are the correlations between METTL3 and different T cell subsets (**A**), different CD4 subsets (**B**), B cells (**C**), and different myeloid cell subsets (**D**), respectively

To further explore the role of METTL3 in the tumor microenvironment, we conducted a correlation analysis between METTL3 expression level and tumor-infiltrating immune cell scores in PAAD patients from the TCGA database. We found that METTL3 was positively correlated with CD4, CD8, γδT, B cells, and negatively correlated with natural Treg (nTreg) cells (Fig. 5A, C), suggesting that the increased expression of METTL3 may play an important role in the regulation of innate as well as adaptive immune response. We further analyzed the relationship between METTLE3 and CD4^+^T cell subsets, and found that METTLE3 was positively correlated with Th1 and Tfh cells, while negatively correlated with Th2 and Th17 cells (Fig. 5B). In regard to the myeloid cell population, our analysis also showed that METTL3 was negatively correlated with DCs, macrophages, and monocytes (Fig. 5D). Thus, all these results showed that METTL3 was associated with a variety of tumor-infiltrating immune cells, but its biological role in different immune cell subsets remains to be further explored.

### Functional enrichment analysis of METTL3 and its highly correlated genes

**Fig. 6:**
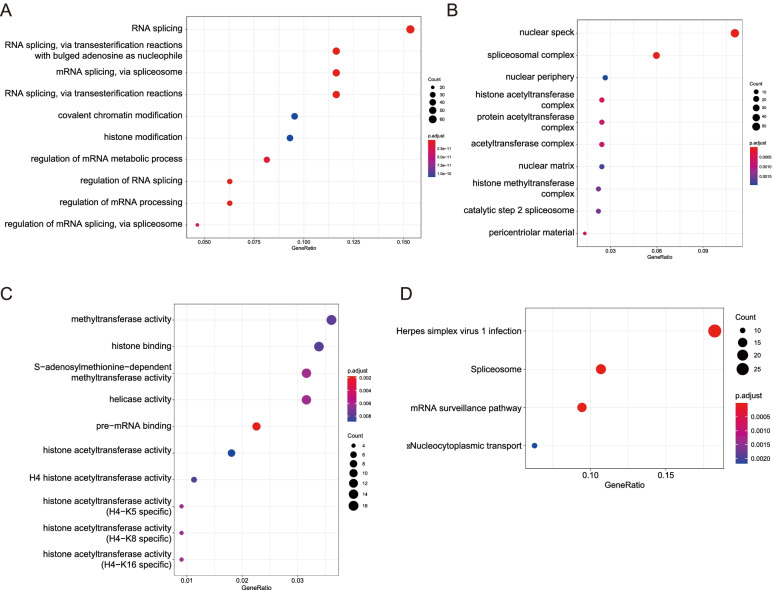
Functional enrichment analysis of METTL3 and its highly correlated genes. Based on the functional enrichment analysis of METTL3 and its highly related genes identified in PC data of GO database (**A–C**) and KEGG database (**D**), biological processes with *r* > 0.5 were selected for display. Among them, **A–C** are biological processes such as BP, CC, and MF, respectively

We then performed a functional enrichment analysis based on METTL3 and its highly correlated genes identified in PC data from the GO database: the top 3 enriched BP processes were “RNA splicing”, “RNA splicing, via transesterification reactions with bulged adenosine as nucleophile”, “mRNA splicing, via spliceosome” (Fig. 6A); the top 3 enriched CC processes were “nuclear speck”, “spliceosomal complex”, “nuclear periphery” (Fig. 6B); the top 3 enriched MF processes were “methyltransferase activity”, “histone binding”, “S-adenosylmethionine-dependent methyltransferase activity” (Fig. 6C). The results of the analysis in the KEGG database found that METTL3 was associated with processes such as “Herpes simplex virus 1 infection”, “Spliceosome”, and “mRNA surveillance pathway”, as shown in Fig. 6D. It was shown that METTL3 might be involved in RNA splicing and methylation modification in PC.

## Discussion

In our present study, the TCGA, GTEx, and GEO public databases were used to study the mRNA expression level of the m^6^A family members and its relationship among PC tissues and normal pancreatic tissue. The immunohistochemistry was used to analyze the difference of METTL3 expression between cancer tissues and adjacent normal tissues. Based on the analysis of different databases, we found that the m^6^A enzyme system showed high correlations with PC progression, and METTL3 mRNA expression level in PC tissues was significantly higher than that in adjacent normal tissues. Moreover, scRNA-seq data indicated that METTL3 showed a higher expression level in malignant epithelial cells. Our immunohistochemistry results also confirmed that the intensity of METTL3 immunostaining in PC tissues was significantly higher than that in adjacent normal tissues. The OS of PC patients with high expression of METTL3 protein was significantly poorer than those with low expression of METTL3 protein. Further analysis from the database showed that METTL3 expression was associated with a variety of tumor-infiltrating immune cells and was involved in m^6^A modification and metabolism in PC tissues.

Based on the analysis of different databases, we found that the m^6^A enzyme system showed high correlations with PC progression. First, we found that at the mRNA level, METTL3 expression is significantly higher in cancer tissues compared with that in adjacent normal tissues, for example, in PC or colon cancer. Second, we also performed the tissue microarray and IHC to detect the expression of METTL3 at the protein level, which was confirmed significantly higher in cancer tissues than that in the corresponding adjacent normal tissues. And our results showed that the high expression level of METTL3 protein in human PC tissues was correlated with poor OS of the patients. Moreover, the protein level of METTL3 was significantly correlated with age and tumor size. The COX model analysis showed that the expression of METTL3 and tumor diameter could be used as independent risk factors for prognostic prediction of the PC patients. Therefore, we herein reported that the METTL3 expression was significantly increased in PC tissues, and higher expression of METTL3 was a poor prognostic factor for the prognosis prediction of PC patients. Consistent with our results, it has been demonstrated that METTL3 is upregulated in cancer tissues compared with the adjacent normal tissues in human breast cancer and gastric cancer, and it also can be used as a candidate target for individualized treatments against these malignancies [13, 25].

Recently, it has been paid close attention to the regulation of m^6^A methylation in cancer cells on the efficacy of immune checkpoint blockade therapy against cancer [26–28]. For example, the depletion of METTL3 and METTL14 could significantly increase the efficacy of anti-PD-1 therapy against colorectal cancer and melanoma [26]. And in mechanism, IFN-γ-Stat1-Irf1 signaling was activated after the depletion of METTL3 and METTL14, leading to the increased secretion of IFN-γ, and finally enhanced the CD8^+^T cell-mediated anti-tumor response in the tumor microenvironment [26]. Herein, according to the database analysis, we also found that METTL3 in PC cancer cells was correlated with a variety of tumor-infiltrating immune cells. Therefore, whether the operation of METTL3 in PC cells could increase the sensitivity of this malignancy in response to immune checkpoint blockade therapy still remains to the elucidated.

## Conclusion

Taken together, our study demonstrated that the METTL3 expression was significantly increased in PC tissues, and it could be used as an important prognostic factor for this malignancy.

## Data Availability

The analyzed data sets generated during the present study are available from the corresponding author on reasonable request.
